# Supplementary material for: Novel fragile X syndrome 2D and 3D brain models based on human isogenic FMRP-KO iPSCs
Source: Cell Death Dis. 2021 May 15;12(5):498. doi: 10.1038/s41419-021-03776-8 (PMC8124071; doi:10.1038/s41419-021-03776-8)
Supplement: Supplementary file 1 — Suppplementary Figure Legends [file 41419_2021_3776_MOESM1_ESM.docx]

***SUPPLEMENTARY MATERIAL***

***Supplementary Figure S1.***

*(A) Screenshot of the UCSC genome browser (http://genome.ucsc.edu; UCSC Genome Browser assembly ID: hg38) showing the human FMR1 locus and the gene models included in the GENCODE track (v32 release). The track includes both protein-coding genes and non-coding RNA genes.*

***Supplementary Figure S2.***

*Scheme illustrating the timeline of the protocols used for generating 2D cortical cultures (A) and 3D brain organoid (B).*

***Supplementary Figure S3.***

*Higher magnification images showing FMRP-WT (top) and FMRP-KO (bottom) neurons within 2D cortical cultures positive for glutamatergic pre-synaptic marker VGLUT1 (red) and post-synaptic marker PSD95 (white) as well as pan-neuronal marker MAP2 (green) at day 54. Scale bar: 10 μm.*

***Supplementary Figure S4.***

*(A) Immunostaining for GFAP (green) and S100β (red) revealing partial co-expression of GFAP and S100β markers in FMRP-WT and FMRP-KO neurons at day 70. Reactivity for S100β, is not exclusive for GFAP-positive astrocytes. Scale bar: 20 μm. (B) Higher magnification images showing FMRP-WT (top) and FMRP-KO (bottom) neurons within 2D cortical cultures positive for glutamatergic pre-synaptic marker VGLUT1 (red) and post-synaptic marker PSD95 (white) as well as pan-neuronal marker MAP2 (green) at day 70. Scale bar: 10 μm.*

***Supplementary Figure S5.***

*Schematic illustration of calcium imaging data processing performed in MATLAB environment: data collected were analyzed through a custom-made algorithm designed to recognize cells, sort calcium events, and select neuronal traces discarding traces characterized by slow rise time typical of astrocytes. Firing rate, amplitude and synchrony of the network were exported in Microsoft Excel to perform final statistics.*

***Supplementary Figure S6.***

*(A) Representative low magnification images of a FMRP-WT brain organoid at day 50 revealing a spread distribution of TBR1 (red) and CTIP2 (green) positive neurons together with the pan-neuronal marker MAP2 (white). Nuclei were stained with DAPI (blue). Scale bar: 500 μm. (B) Higher magnification of a cortical plate region at day 50 positive for TBR1 (red), CTIP2 (green) and MAP2 (white). Nuclei were stained with DAPI (blue). Scale bar: 50 μm. (C) Higher magnification of a cortical plate region at day 100 positive for GFAP (red) and CTIP2 (green). Nuclei were stained with DAPI (blue). Scale bar: 50 μm.*

***Supplementary Figure S7.***

*(A) Representative immunostaining at day 70 for pan-neuronal markers TUJ1 (white) in FMRP-WT and FMRP-KO brain organoids together with intensity quantification (B) of fluorescence signal (p=0.08, MW test; WT n=42/7/2, KO n=29/6/2, slices/organoids/batches). (C) Representative immunostaining at day 100 for pan-neuronal markers TUJ1 (white) in FMRP-WT and FMRP-KO brain organoids together with intensity quantification (D) of fluorescence signal (p=0.1, MW test; WT n=24/4/1, KO n=32/4/1 slices/organoids/batches).*
