# Supplementary figures and images for: Novel fragile X syndrome 2D and 3D brain models based on human isogenic FMRP-KO iPSCs
Source: Cell Death Dis. 2021 May 15;12(5):498. doi: 10.1038/s41419-021-03776-8 (PMC8124071; doi:10.1038/s41419-021-03776-8)

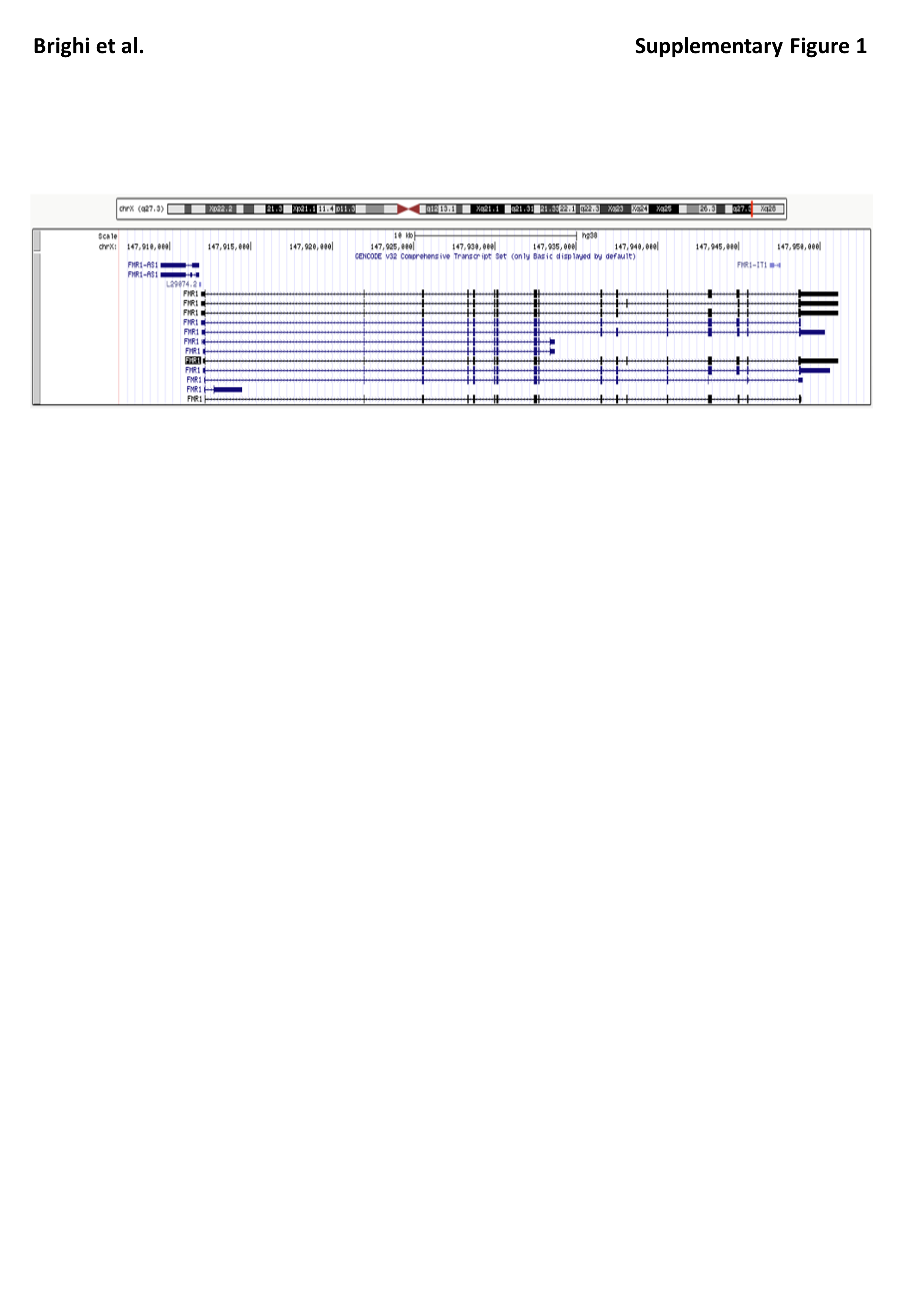

Supplement: Supplementary file 2 — Supplementary Figure S1 [file 41419_2021_3776_MOESM2_ESM.tif]

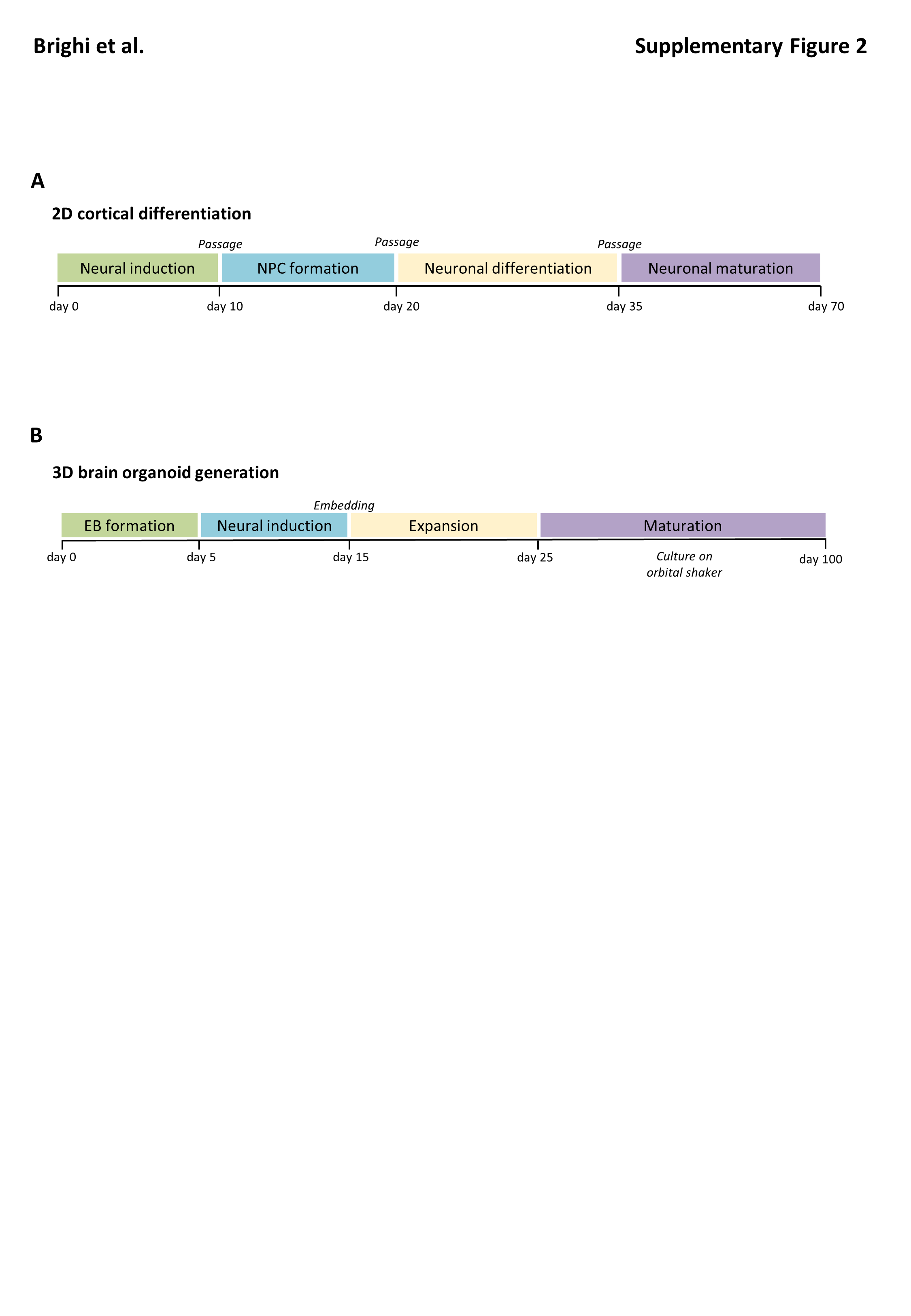

Supplement: Supplementary file 3 — Supplementary Figure S2 [file 41419_2021_3776_MOESM3_ESM.tif]

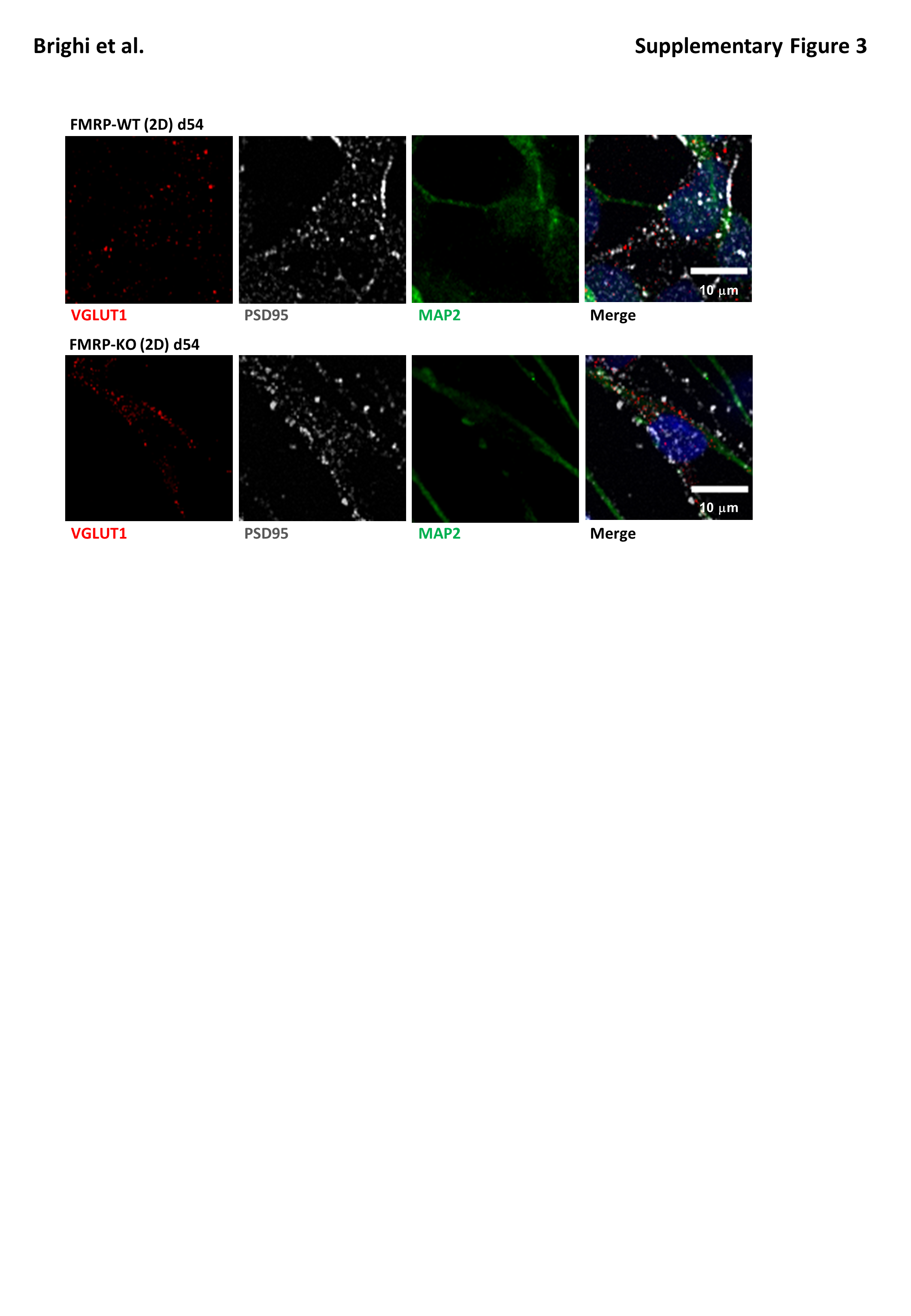

Supplement: Supplementary file 4 — Supplementary Figure S3 [file 41419_2021_3776_MOESM4_ESM.tif]

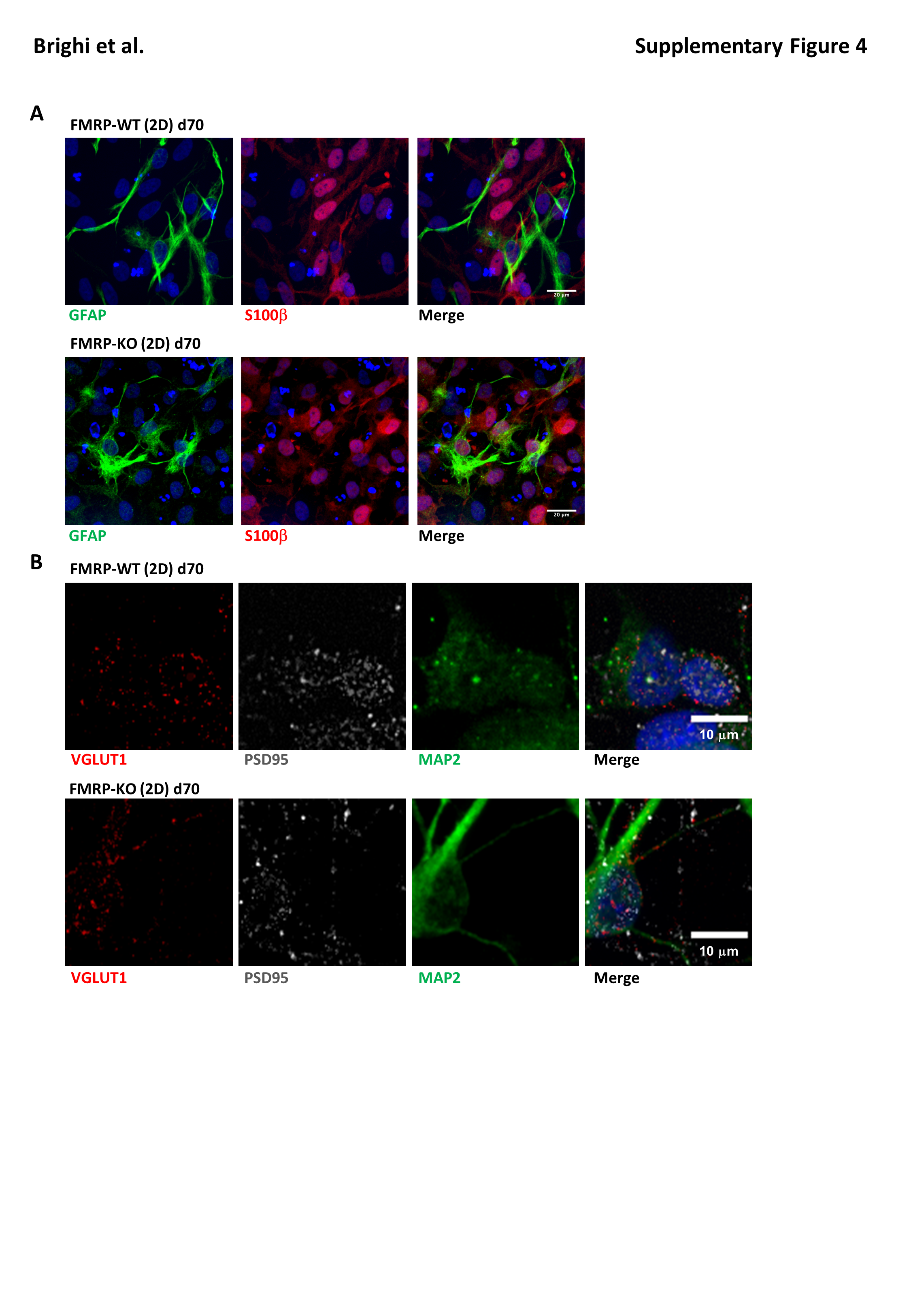

Supplement: Supplementary file 5 — Supplementary Figure S4 [file 41419_2021_3776_MOESM5_ESM.tif]

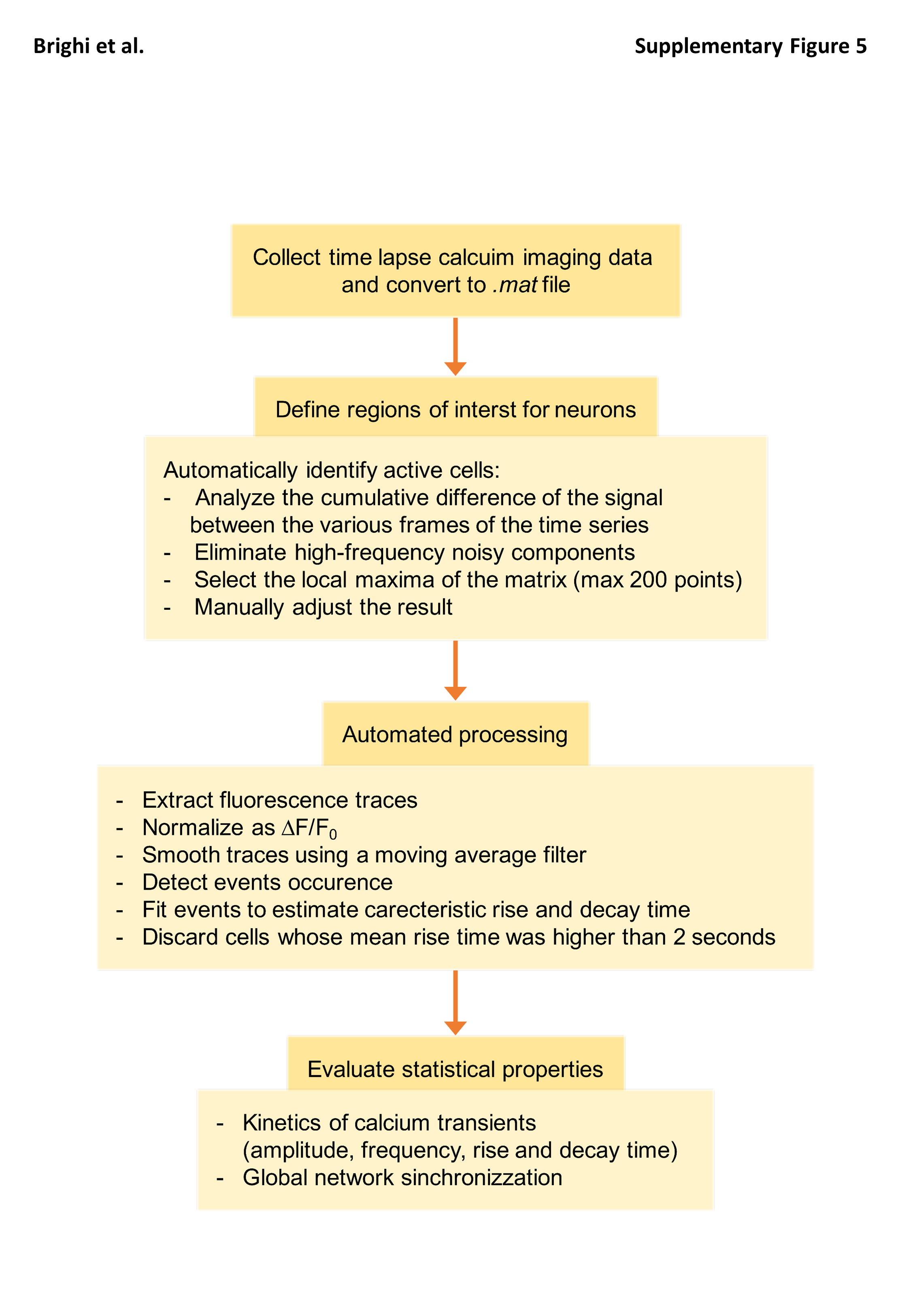

Supplement: Supplementary file 6 — Supplementary Figure S5 [file 41419_2021_3776_MOESM6_ESM.tif]

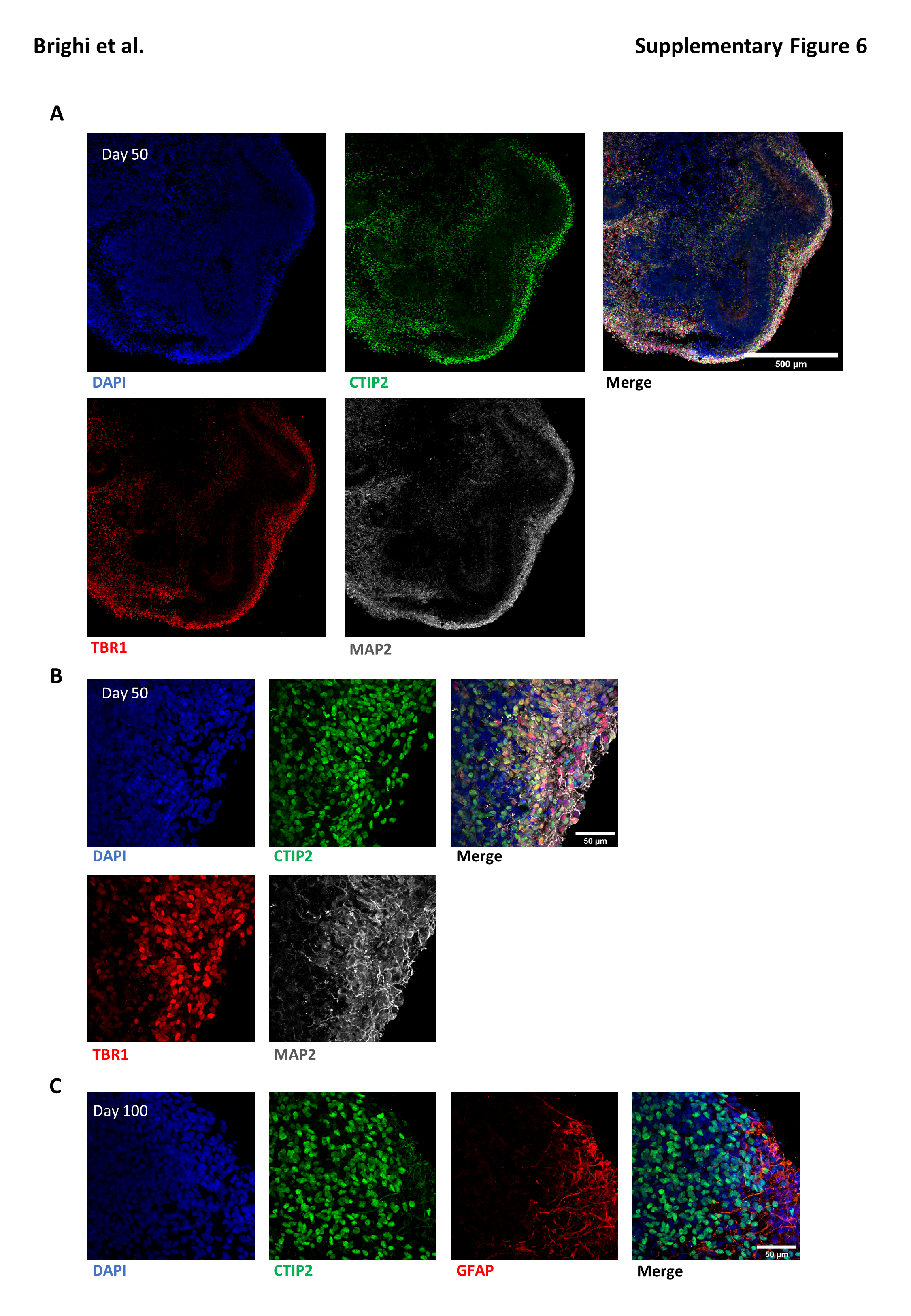

Supplement: Supplementary file 7 — Supplementary Figure S6 [file 41419_2021_3776_MOESM7_ESM.tif]

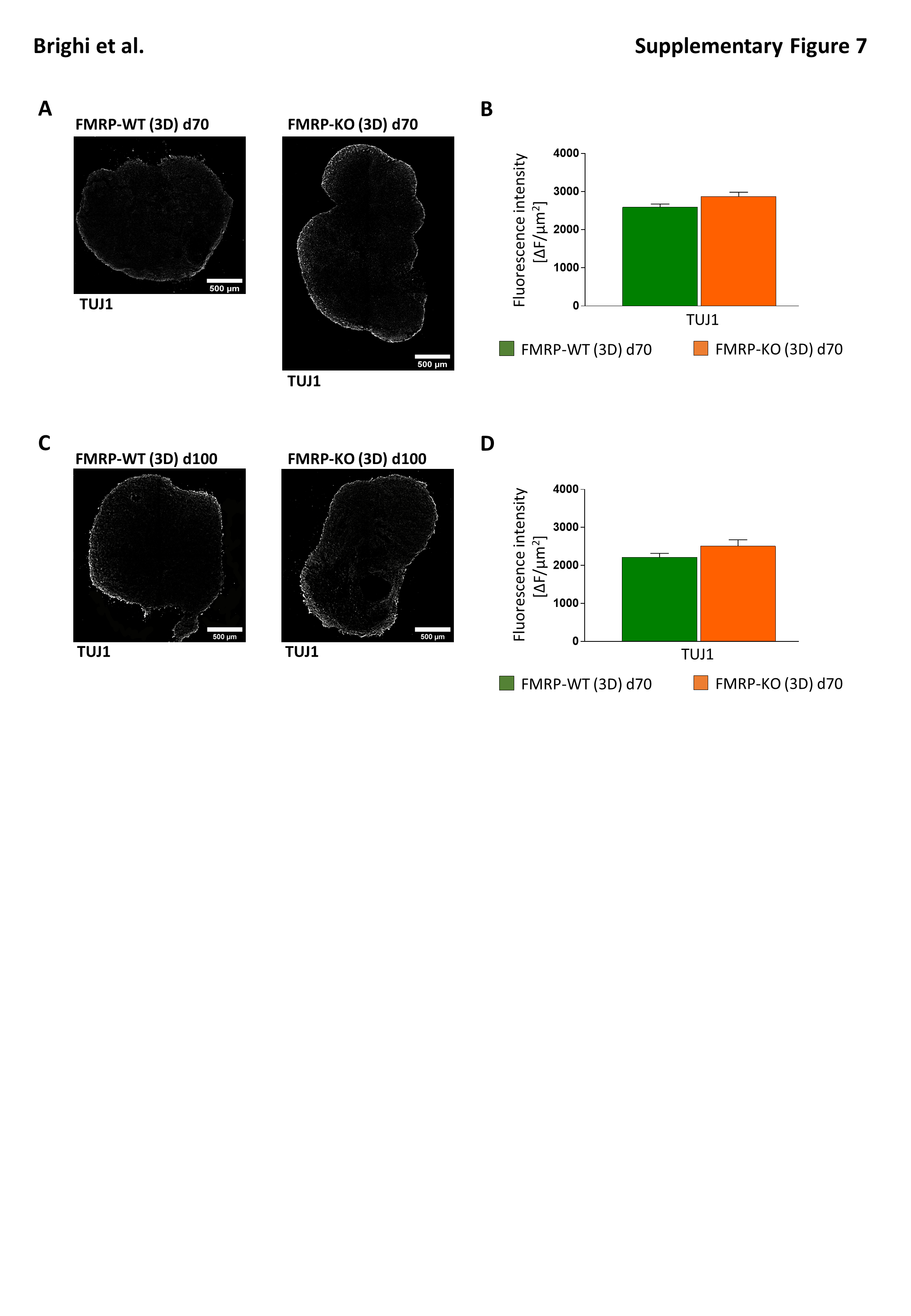

Supplement: Supplementary file 8 — Supplementary Figure S7 [file 41419_2021_3776_MOESM8_ESM.tif]

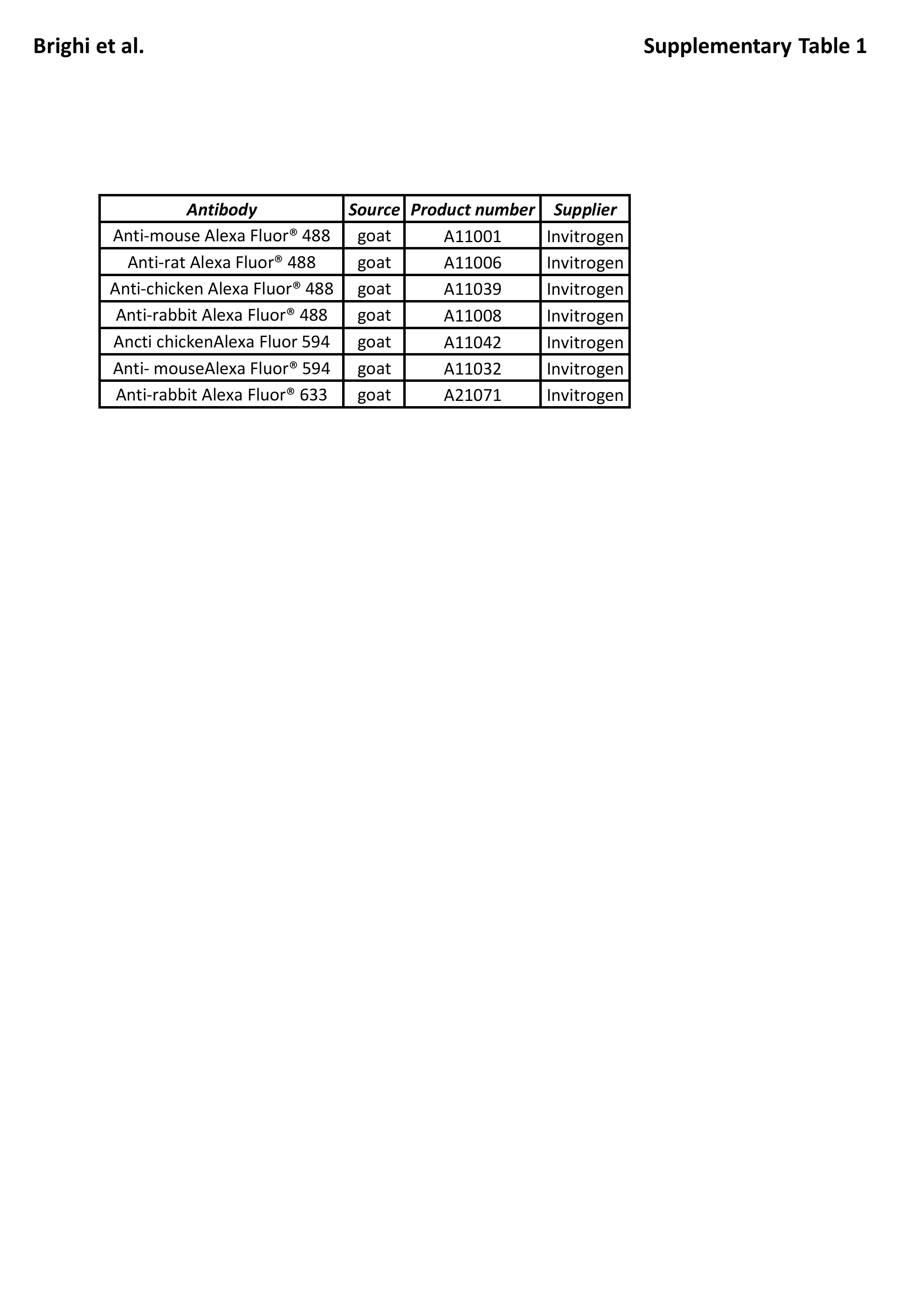

Supplement: Supplementary file 9 — Supplementary Table1 [file 41419_2021_3776_MOESM9_ESM.tif]

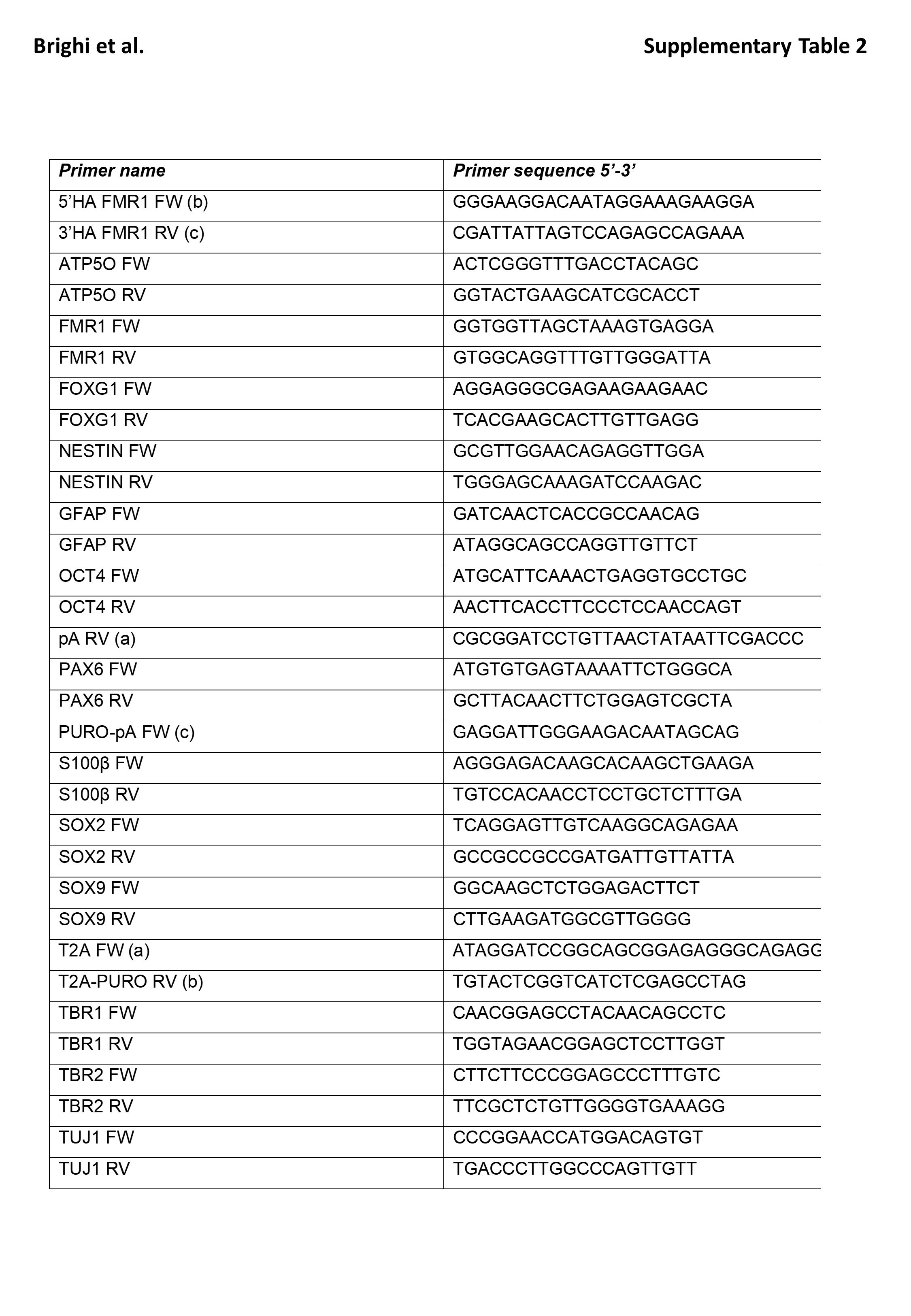

Supplement: Supplementary file 10 — Supplementary Table2 [file 41419_2021_3776_MOESM10_ESM.tif]
